# Gestational Age Assessment in the Ghana Randomized Air Pollution and Health Study (GRAPHS): Ultrasound Capacity Building, Fetal Biometry Protocol Development, and Ongoing Quality Control

**DOI:** 10.2196/resprot.3797

**Published:** 2014-12-18

**Authors:** Ellen A Boamah, KP Asante, KA Ae-Ngibise, Patrick L Kinney, Darby W Jack, Grace Manu, Irene T Azindow, Seth Owusu-Agyei, Blair J Wylie

**Affiliations:** ^1^Kintampo Health Research CentreBrong Ahafo Region, KintampoGhana; ^2^Mailman School of Public Health, Columbia UniversityDepartment of Environmental Health SciencesNew York, NYUnited States; ^3^Massachusetts General HospitalDivision of Maternal-Fetal Medicine, Department of Obstetrics and GynecologyHarvard Medical SchoolBoston, MAUnited States

**Keywords:** ultrasound, capacity building, gestational age, biometry, household air pollution

## Abstract

**Background:**

Four million premature deaths occur yearly as a result of smoke from cooking fires. The Ghana Randomized Air Pollution and Health Study (GRAPHS) is underway in the Kintampo North municipality and South district of rural Ghana to evaluate the impact of improved cook stoves introduced during pregnancy on birth weight and childhood pneumonia. These hypotheses are being tested in a cluster-randomized intervention trial among 1415 maternal-infant pairs within 35 communities assigned to a control arm (traditional cooking) or one of two intervention arms (cooking with an improved biomass stove; cooking with liquefied petroleum gas stoves).

**Objective:**

The trial is designed to ensure delivery of the stove intervention prior to the period of maximal fetal growth. To answer questions about the impact of household air pollution on pregnancy outcome, accurate gestational age assessment is critical. This manuscript describes in detail the development of the gestational dating protocol, intensive ultrasound training involved, ultrasound capacity building, and ultrasound quality control program.

**Methods:**

Ultrasound training occurred in several phases over the course of 2 years. Training included a basic obstetric ultrasound course offered to all midwives performing antenatal care at the two study hospitals, followed by a more intense period of hands-on training focused on fetal biometry for a select group of providers demonstrating aptitude in the basic course. A standard operating procedure was developed describing how to obtain all fetal biometric measurements. Consensus was obtained on how biometric images are used in the trial to establish gestational age and estimate the delivery date. An ongoing ultrasound quality control program including the use of an image scorecard was also designed.

**Results:**

Publication of trial results is anticipated in late 2016.

**Conclusions:**

Use of ultrasound should be strongly considered in field-based trials involving pregnant women to accurately establish gestational age, as menstrual dates may be incorrect or unknown. The inclusion of ultrasound in areas where ultrasound capacity does not previously exist requires a significant investment of time and resources. Such investment ensures appropriate training, high quality images, and accurate dating pregnancies. We outline our ultrasound training, image acquisition, quality control, and dating protocols in detail.

**Trial Registration:**

Clinicaltrials.gov NCT01335490; http://clinicaltrials.gov/ct2/show/NCT01335490 (Archived by WebCite at http://www.webcitation.org/6UbERJNO6).

## Introduction

### Background: The Ghana Randomized Air Pollution and Health Study (GRAPHS)

The majority of households in sub-Saharan Africa rely on solid biomass fuels such as charcoal, wood, or crop residues for cooking. Women, as the primary cooks, and their young children playing nearby are particularly vulnerable to harmful pollutants released from the inefficient combustion of these fuels. The resulting household air pollution causes nearly 4 million premature deaths per year and represents one of the major global environmental risk factors for reduced life expectancy, due in large part to an increased risk of death from acute lower respiratory infections (pneumonia) in childhood [1-3]. The overall objective of the Ghana Randomized Air Pollution and Health Study (GRAPHS; Trial Registration NCT01335490) is to test the effectiveness of improved cook stoves introduced prior to the third trimester in pregnancy to increase birth weight and reduce pneumonia in the first year of life (Jack et al, forthcoming). If effective, distribution of cleaner cooking technology through existing antenatal programs may be considered by policymakers to reduce household air pollution attributable deaths in early childhood.

Our hypotheses are being tested in a cluster-randomized intervention trial among 1415 maternal-infant pairs in the Kintampo North municipality and South district of rural Ghana in West Africa. In the 35 trial communities, pregnant women are being identified via community-based fieldworkers, taking advantage of an existing health and demographic surveillance system. Women confirmed by ultrasound to be carrying a single live fetus less than 24 weeks estimated gestation who consent to participation are enrolled into the trial. Enrolled women are followed during pregnancy and their children monitored through one year of life to assess the impact of the intervention on two primary endpoints (birth weight and acute lower respiratory infections). Within intervention communities, improved cook stoves are distributed to enrolled pregnant women prior to 28 weeks, along with health insurance and an insecticide-treated bed net. Women in one of the intervention arms will receive a two-burner liquefied petroleum gas (LPG) stove along with LPG, which will be supplied to them throughout the study duration. In the other arm, enrolled women receive two improved biomass stoves (BioLite, Brooklyn, New York). In laboratory settings, LPG and the BioLite stoves achieve reductions of 80% or more in combustion products. The impact in field settings from exposure reduction on health outcomes using these stove interventions has not been established.

### Rationale for Accurate Gestational Age Assessment in GRAPHS

To ensure that our stove interventions are delivered prior to the third trimester when fetal growth is at a maximum, accurate gestational age assessment of the screened pregnant women is required. Furthermore, if we identify a difference in mean birth weight by intervention arm, we aim to determine whether this stems from a difference in infants born too early, too small, or both. These secondary trial outcomes (preterm birth and small for gestational age) also require knowledge of the gestational age. In the first half of pregnancy, ultrasound evaluation of gestational age is more accurate than menstrual dating [4-5]. Postnatal estimates of gestational age, and examinations such as the Ballard [6] or Dubowitz [7], are unable to verify inclusion criteria and may misclassify preterm infants as term or small for gestational infants as appropriately grown [8]. Menstrual recall is imperfect in our population. We therefore chose to incorporate ultrasound into GRAPHS at the time of enrollment using midwives at the study hospital. The feasibility of training mid-level providers to perform fetal biometry in resource-limited settings has been previously demonstrated [8-10]. In this manuscript, details of the ultrasound training program, the protocol for gestational age assessment in GRAPHS, and the ongoing quality review process are described. This will clarify how gestational age is determined for the trial and may be of utility to others embarking on similar efforts.

## Methods

### Ethics Approval and Funding

Ethical approvals for GRAPHS were obtained from the Institutional Review Boards of Columbia University Medical Center and the Massachusetts General Hospital/Partners Healthcare, the Ghana Health Service Ethical Review Committee, and the Kintampo Health Research Centre Institutional Ethics Committee. Funding for the trial is provided through the United States National Institute of Environmental Health Sciences (NIH 1R01ES019547) and the Ghana Ministry of Health.

### Study Area

GRAPHS is taking place in 35 randomly selected communities in the Kintampo North Municipality and South Districts, which are located in the Brong Ahafo Region of Ghana. The two districts are largely rural and are monitored by an extensive health and demographic surveillance system [11]. Neonatal mortality is 32 deaths per 1000 live births and infant mortality estimated at 52 deaths per 1000 live births [12]. Antenatal attendance among pregnant women is high, with more than 95% attending at least once [13].

### Ultrasound Equipment

Three portable SonoSite S180 machines equipped with curved 5-2MHz C60 transducers (SonoSite, Inc, Bothell, Washington) were purchased for image acquisition in GRAPHS. The S180 machine was initially developed as a portable point-of-care ultrasound machine for the US Department of Defense for use on the battlefield and, as such, offers durability and robustness atypical of many other machines.

### Ultrasound Training

Ultrasound training occurred in several phases over 2 years as summarized in Table 1. We targeted midwives for ultrasound training rather than ultrasound technicians so that acquired skills could elevate obstetric care at participating hospitals. Phase 1 consisted of a 1-week introductory course in basic obstetric ultrasound that was developed and led by the GRAPHS obstetrician (BJW). All 15 midwives that provided antenatal care at either Kintampo North Municipal Hospital in Kintampo or Kintampo South District Hospital in Jema participated. Topics covered included assessment of viability, location of the pregnancy, placental location, plurality of the gestation, and a basic introduction to fetal biometry. Didactics were combined with supervised practice. A special emphasis was placed on how to turn on and use the ultrasound machines located at each hospital so that basic point-of-care ultrasound could be incorporated as needed when formal ultrasound at the hospital was unavailable. Participating midwives received a certificate of attendance in Basic Obstetric Ultrasound following this course of instruction.

**Figure 1:**
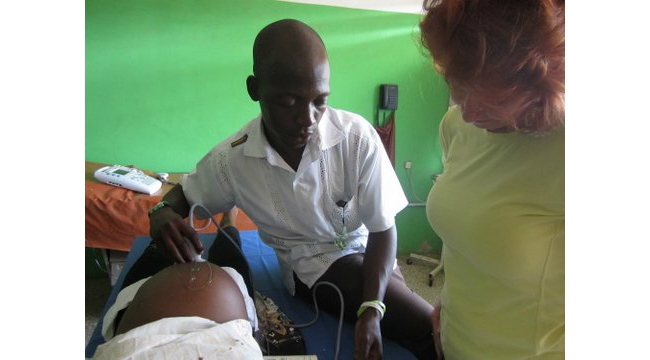
Hands-on ultrasound training of Ghanaian midwives by US-based registered diagnostic medical sonographer.

A US-based registered diagnostic medical sonographer travelled to Kintampo to conduct Phase 2 of the ultrasound training during May 2012. A group of five midwives and one medical assistant from the two district hospitals were selected to undergo advanced training in this intensive hands-on course based on their performance in the first phase of training. Participants were instructed in the acquisition of fetal biometric measurements including first trimester measurements such as crown-rump length (CRL) and second trimester measurements such as biparietal diameter (BPD) and femur length (FL). Didactics emphasized identifying appropriate landmarks, ensuring adequate magnification of images, and placing calipers in the correct location. All training materials were developed by the study obstetrician using standard obstetric ultrasound textbooks. Sessions were devoted to practicing fetal biometry on volunteer subjects with immediate feedback and tips provided by the US sonographer (Figure 1). Additional practice was assigned upon identification of specific deficiencies.

Of the six midwives that underwent the intensive Phase 2 training, four became GRAPHS sonographers based on their aptitude, interest, and anticipated availability for the duration of the study. Additional supervised practice in fetal biometry for these four midwives (Phase 3) occurred through the Alliance for Maternal and Newborn Health Improvement Study (AMANHI; Clinical Trials Registration NCT01699945). In AMANHI, 1000 pregnant women underwent ultrasound examinations at less than 20 weeks in Kintampo. GRAPHS sonographers were paired with two US maternal-fetal medicine fellows to perform the AMANHI ultrasound scans. This afforded the GRAPHS sonographers the opportunity to solidify their scanning skills with an immediately available experienced teacher.

During the second half of 2013, all GRAPHS activities with the exception of the intervention were piloted in a cohort of 50 pregnant women. Ultrasound images obtained in the course of the pilot were saved in a de-identified form and transferred electronically to the GRAPHS obstetrician for remote review and feedback constituting Phase 4 of ultrasound training.

**Table 1:** Phases of ultrasound training in GRAPHS^a^.

Phase of training	Trainer	Participants	Length of training	Topics covered
Phase 1	Trial obstetrician-perinatologist	All midwives (15) performing antenatal care at study district hospitals	1 week	Didactics: Basic obstetric ultrasound including assessment of viability, location of the pregnancy, placental location, plurality of the gestation, introduction to fetal biometry
				Hands-on: viability, pregnancy location, plurality, placental location
Phase 2	US-based registered diagnostic medical sonographer	5 midwives and 1 medical assistant, selected after Phase 1 for demonstration of ultrasound aptitude	2 weeks	Didactics: Focus on fetal biometry (CRL, BPD, FL^b^), appropriate landmark identification, adequate image magnification, correct caliper placement
				Hands-on: Fetal biometry all trimesters
Phase 3	US-based maternal-fetal medicine fellows	4 midwives selected after Phase 2 for aptitude and interest in ultrasound plus anticipated availability for trial duration	3 months	Hands-on: Fetal biometry <20 weeks gestation; practice scanning in 1000 subjects enrolled into separate maternal child health study
Phase 4	Trial obstetrician-perinatologist	4 study sonographers trained in Phase 3	6 months	Hands-on: Image acquisition of pilot GRAPHS participants; image review and written feedback by study obstetrician-perinatologist

^a^GRAPHS: Ghana Randomized Air Pollution Health Study

^b^CRL: crown-rump length; BPD: biparietal diameter; FL: femur length

### Ultrasound Protocol in GRAPHS

#### Overview

GRAPHS employs resident field workers in all 35 clusters. These field workers identify pregnant women for potential trial participation. Interested subjects are referred to the Kintampo Municipal and Kintampo South District Hospitals for completion of screening.

#### Basic Overview Scan

At the screening visit, the study midwife equipped with a portable ultrasound machine, ultrasound transmission gel, tissue, as well as the ultrasound gestational age form performs the screening ultrasound examinations on all referred pregnant women. A screening patient identification number is generated by the hospital-based field worker and provided to the research midwife for entry into the ultrasound machine so no personal identifying information is recorded or stored with the ultrasound images. The entirety of the abdomen is scanned to confirm pregnancy, to identify the location, and to verify a single live fetus (Table 2). If the pregnancy is too early to be visible by ultrasound, warning signs of an ectopic gestation are reviewed and a follow-up ultrasound rescheduled in 1 to 2 weeks. If twins or higher order multiples are discovered or if the fetus is not alive, the scan is concluded, the woman is deemed ineligible for trial participation, and she is referred for antenatal care with a notation made of the scan’s findings. Biometric measurements are obtained for those still eligible after this overview scan (described below). All women receive a printed ultrasound image of their baby.

**Table 2:** Components of the overview scan in GRAPHS^a^.

Components of overview scan	Comments
Determine if subject pregnant	If no fetal pole visible within uterus, repeat ultrasound scheduled in 1-2 weeks; ectopic precautions reviewed
Establish location of pregnancy	If extrauterine location suspected, woman referred urgently for care at hospital
Determine plurality of gestation	Women carrying more than one fetus referred for routine antenatal care
Evaluate viability of pregnancy	If no fetal heart motion detected, repeat ultrasound scheduled if ≤10 weeks; if >10 weeks, referred to hospital for evaluation of potential missed abortion

^a^GRAPHS: Ghana Randomized Air Pollution Health Study

#### Fetal Biometry

##### First Trimester Biometry (<14 Weeks Gestation)

**Figure 2:**
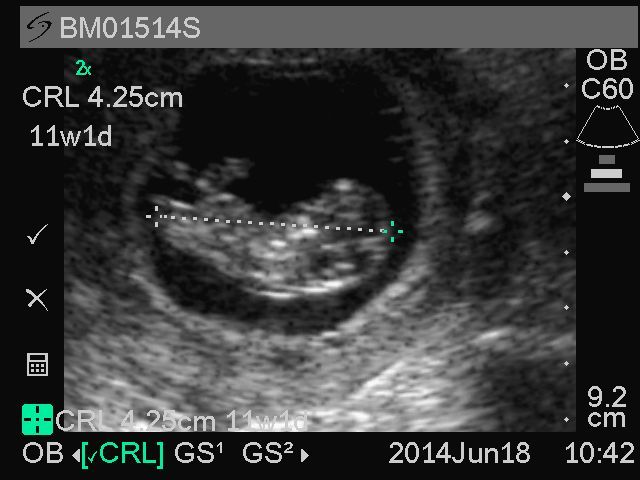
Representative sample image of crown-rump length from Ghana Randomized Air Pollution and Health Study (GRAPHS) participant.

For first trimester scans, midwives are instructed to obtain CRL measurements for pregnancies less than 14 weeks. When both the crown and rump are visible on the screen in the approximate midsagital plane imaged horizontally on the ultrasound screen, the image is zoomed to occupy more than 50% of the screen and frozen. Calipers are placed at the exterior edge of the skull (the crown) and at the inferior aspect of the pelvic bones (the rump). A sample GRAPHS CRL image is shown in Figure 2. The measured image is then saved to the portable ultrasound machine. A total of three CRL images are obtained. A summary report is generated by the ultrasound machine, which averages the three saved images to estimate an overall gestational age and corresponding estimated date of delivery. Both the measurements and the corresponding gestational age estimated by the machine software are recorded onto the Ultrasound Gestational Age Form. The machine-generated delivery date is considered the Working Estimated Delivery Date for the trial and is used to schedule all other trial activities and to anticipate the delivery.

##### Second Trimester Biometry (14-24 Weeks Gestation)

**Figure 3:**
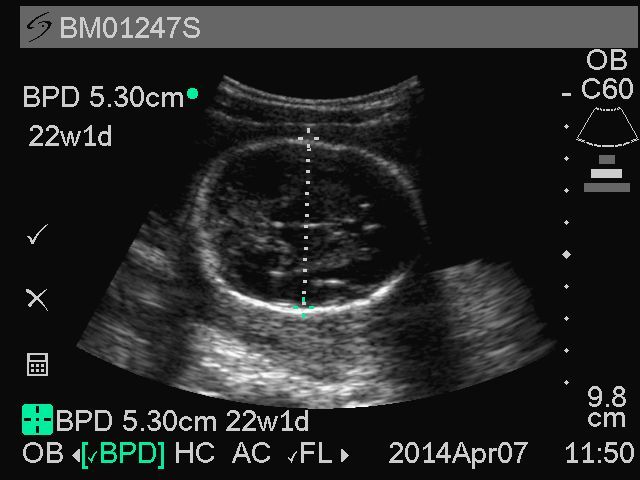
Representative sample of biparietal diameter from Ghana Randomized Air Pollution and Health Study (GRAPHS) participant.

For second trimester scans, midwives are instructed to obtain BPD and FL measurements for pregnancies 14 weeks gestation or more (Table 3). The abdominal circumference is not used in GRAPHS as the majority of subjects are screened prior to 20 weeks when this biometric measurement is less relevant for gestational age determination. For fetuses measuring 14 weeks by BPD, additional CRL images are obtained to help verify the gestational age. To obtain a quality BPD image, study midwives are instructed to identify the fetal head, orienting the transducer so that the skull is imaged side to side on the screen, is visible as a continuous echogenic line, and appears oval rather than round in shape. The thalamus (butterfly appearance) and the cavum septum pellucidum (looks like an equal sign) are then identified to find the appropriate plane and the image zoomed to occupy more than 50% of the screen prior to freezing. The calipers are then placed at the widest portion of the skull from outer-to-inner. The top caliper is placed in contact with the external surface of the echogenic skull line and the bottom caliper placed along the inner surface of the echogenic skull line on the lower portion of the image. The measurement line intersects the longitudinal axis of the skull at a right angle. A representative BPD image from GRAPHS is shown in Figure 3. The measured image is then saved to the portable ultrasound machine. A total of three BPD images are obtained.

**Figure 4:**
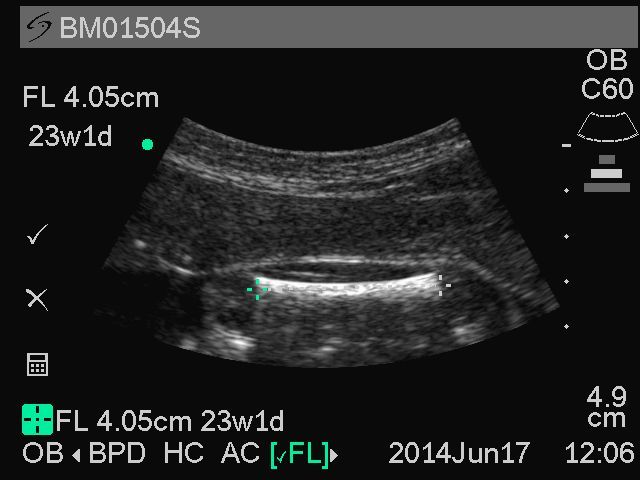
Representative sample image of femur length from Ghana Randomized Air Pollution and Health Study (GRAPHS) participant.

To obtain a quality FL image, study midwives are instructed to scan axially from the fetal abdomen toward the fetal pelvis until the bladder is identified. The echogenic area closest to the skin outside the pelvis is the femur in cross-section. Turning 90 degrees on this echogenic circle elongates the femur into view. The transducer is oriented so that the full extent of the femur is visible side to side on the screen and appears straight rather than curved. Calipers are placed at the external edges of the femur bone without inclusion of the secondary ossification center. A representative image from GRAPHS is shown in Figure 4. By scanning through the abdomen to the pelvis rather than randomly looking for fetal bones, the femur rather than the humerus is identified. The measured image is then saved to the portable ultrasound machine. A total of three FL images are obtained.

The ultrasound machine averages the three BPD and three FL measurements to estimate an overall gestational age and corresponding estimated date of delivery. Both the measurements and the corresponding gestational age estimated by the software are recorded onto the Ultrasound Gestational Age Form**.** The machine-generated delivery date serves as the Working Estimated Delivery Date for the trial and is used to schedule all other trial activities and to anticipate the delivery. Menstrual dates are not used.

#### Transfer of Images

Images are transferred at the end of each day from the portable ultrasound machine to a password-protected computer in Kintampo and backed up to a secure server accessible by both the US- and Ghana-based researchers. Every 2 weeks, images are transferred electronically for review by the study obstetrician.

#### Gestational Age Assessment

The two best images for each biometric parameter are selected and averaged by the study obstetrician blinded to intervention arm. Should more than one image be deemed substandard, the single best image will be used. If all three images are unacceptable, research staff will be instructed to have the subject return for a second dating scan as soon as possible.

For pregnancies <14 weeks gestation, the average CRL measured is used to date the pregnancy (Table 3). Pregnancies measured in the 14^th^ and 15^th^ week are dated by the BPD only. After 16 weeks, the selected BPD and FL measurements are then averaged to estimate the overall gestational age. The Trial Estimated Date of Delivery is then calculated using an electronic wheel, available as an app for download on mobile phones, and recorded in the Ultrasound Summary Report (Multimedia Appendix 1) [14]. As noted previously, the Working Estimated Date of Delivery will be used by research personnel to time research activities and plan for the anticipated birth. However, the Trial Estimated Date of Delivery will be the due date used in the analysis phase of GRAPHS to determine gestational age at enrollment and at delivery. This allows for removal of any images not meeting study standards.

While the ultrasound scan included in the GRAPHS trial is not intended as a screen for fetal anatomy, the study obstetrician reports any anomalies suspected in the images she reviews to the trial supervisor. These women are referred for formal ultrasound with notations as to the suspected anomaly. Similarly, any abnormalities suspected by the study midwives are transferred to the study obstetrician for more urgent review.

**Table 3:** Biometric parameter used to establish gestational age in GRAPHS^a^.

Gestational age	Biometric parameter used to establish trial estimated delivery date
Less than 14 0/7 weeks gestation	CRL, average of two best images^c^
14 0/7 through 15 6/7 weeks gestation^b^	BPD, average of two best images^c^
16 0/7 weeks gestation and greater	BPD +FL, average of two best BPD and two best FL images^c^

^a^GRAPHS: Ghana Randomized Air Pollution Health Study.

^b^Research midwives are encouraged to obtain both CRL and BPD for pregnancies where gestational age appears to be in the 13-15 week range.

^c^Best images are determined by study obstetrician following review of all images.

#### Ongoing Quality Review

At the time of image review for gestational age assessment, the study obstetrician identifies any image quality concerns that require feedback. Such commentary has included reminders about appropriate zooming of images and gentle admonishments about precise caliper placement. One study sonographer’s employment with the trial was terminated due to an inability to meet quality standards.

In addition to this informal immediate feedback, 5% of all subjects are selected randomly by the trial supervisor for formal quality review by the study obstetrician. Using a set of predefined criteria (Table 4), the scan is formally graded and recorded onto a MS Excel spreadsheet for the sonographer. A maximum of 7 points for each biometric parameter is awarded in the areas of correct magnification, correct plane identification, and correct caliper placements. These formal reviews are shared with the study midwives for performance improvement and kept as part of the study staff’s employee file.

**Table 4:** Quality scorecard for components of fetal biometric measurements.

Quality criteria	Crown rump length 1 point each^a^	Biparietal diameter 1 point each^a^	Femur length 1 point each^a^
**Correct magnification**			
	Good magnification (>50% of image)	Good magnification (>50% of image)	Good magnification (>50% of image)
	Neutral position (not hyperflexed or hyperextended)	--	--
**Correct plane**			
	Fetus horizontal (side to side on screen)	Skull is oval and visible throughout	Femur imaged side to side on screen
	Full extent of crown visible	Thalamus is visible	Only one bone in this portion of the extremity
	Full extent of rump visible	Skull side to side on screen	Upper femur measured
	--	--	Full extent of femur visualized (solid straight line)
**Correct caliper placement**			
	Crown caliper at exterior edge of skull	Calipers placed perpendicular to the long axis of the skull	Calipers placed at edge of echogenic bone (outer to outer)
	Rump caliper placed at lower spine	Top caliper placed on outer portion of skull	Secondary ossification centers not measured
	--	Bottom caliper placed on inner portion of skull	--
Total score	Maximum=7 points	Maximum=7 points	Maximum=7 points

^a^Half-points can be awarded.

### Trial Ultrasound Examinations Through June 2014

Since launch of the trial, 1310 pregnant women have been screened for eligibility with an ultrasound examination through the end of June 2014. Of these scans, 146 women were found to be ineligible for the study based upon findings during the ultrasound. This included 91 women whose pregnancy was found to be greater than 24 weeks by fetal biometry, 38 women found not to be pregnant, 16 women carrying twins, and one woman with a nonviable fetus. In addition, 43 examinations were rescheduled as the pregnancy was too early to confirm and date by ultrasound. Of the 513 births that have occurred among those enrolled in the trial, 3 sets of undiagnosed twins were born. One case of fetal hydrocephalus was discovered during Phase 2 of ultrasound training and a cystic hygroma identified in one of the first trimester screening scans.

## Discussion

### Principal Findings

We have described the extensive training and ongoing quality control program developed to ensure a high caliber of ultrasound examination in GRAPHS currently underway in in rural Ghana. The primary objective of the ultrasound training and continuing review is to optimize gestational age assessment in the trial. Accurate gestational age is required to target intervention deployment prior to 28 weeks and to evaluate whether the stove interventions impact rates of preterm birth (delivery <37 weeks) or small for gestational age (birth weight less than 10^th^ percentile), which are secondary outcomes of the study. Additional benefits of ultrasound at enrollment include verification of other trial inclusion criteria such as a singleton gestation, live fetus, intrauterine location, and comparability of study arms. We also anticipate that ultrasound examination and provision of a photo might increase satisfaction of the participating mother.

An equally compelling rationale underlying our decision to include ultrasound in GRAPHS was the training and capacity building this endeavor would bring to both the Kintampo Health Research Centre and the two hospitals where the ultrasounds are being performed. Ultrasound scans can reveal high-risk situations such as women carrying multiples or anomalous fetuses. Potentially life-threatening pregnancy complications such as ectopic gestation and placenta previa also can be identified. The benefits of identifying complicated pregnancies may be even greater compared to resourced settings as maternal and perinatal mortality and morbidity are much higher in low resource settings [15,16]. Ultrasound machines were previously available at both study hospitals but routinely used only during daytime hours and dependent upon physician or radiology technician availability. Prior to the GRAPHS ultrasound training, none of the midwives who provide the majority of antenatal care had been taught to use the machines or interpret the images. By orienting the group of midwives performing antenatal care at these hospitals to the use of the machine and basic obstetric scanning, it is our hope that point-of-care ultrasound will be embraced by the antenatal providers. Capacity is therefore built both for the research center and for the clinical community.

Several key points bear additional emphasis. Building ultrasound capacity was not an overnight phenomenon. This endeavor required a significant investment of time both by educators and trainees. Ultrasound skills cannot be taught in the classroom. While didactics are useful to identify target landmarks, hands-on practice is required. Obstetric scanning necessitates a spatial awareness and an ability to move a transducer in multiple planes as the fetus is mobile and not always in the same orientation. A certain amount of flexibility and fluidity on the part of the person scanning is required to follow the baby and find the correct planes.

Beyond acquisition of high quality images, accurate gestational age assessment requires a standardized protocol. Specifying a priori which biometric parameter will be used for pregnancy dating at different time points in gestation avoids any suggestion that findings were fit to study hypotheses. A single electronic wheel is being used for all GRAPHS participants as many paper-based pregnancy wheels vary in estimated delivery dates depending on the physical size of the wheel. Verification of the gestational age in GRAPHS is time intensive. The study obstetrician personally reviews every ultrasound image for GRAPHS participants to select those suitable for inclusion in dating the pregnancy. Her clinical career as a maternal-fetal medicine specialist has included the review of over 10,000 ultrasound examinations and she therefore brings considerable experience to the trial. These skills are being transferred over the course of the trial to the Ghanaian research team such that future research trials will have local capacity for gestational age assessment.

### Conclusions

We have demonstrated that it is feasible to obtain ultrasound biometry for the accurate establishment of gestational age in a large field-based trial in rural West Africa, following a significant commitment of time, dedication, and resources to achieve and maintain high quality. We have presented our extensive ultrasound training program, quality control process, and gestational age assessment protocol from the GRAPHS trial in the anticipation that our experience may be of utility to others engaged in obstetric research in resource-limited settings.

## Multimedia Appendix 1

Representative sample of Ultrasound Summary Report.

## Abbreviations

AMANHI: Alliance for Maternal and Newborn Health Improvement Study
BPD: biparietal diameter
CRL: crown-rump length
FL: femur length
GRAPHS: Ghana Randomized Air Pollution Health Study
LPG: liquefied petroleum gas
