# Supplementary material for: Gestational Age Assessment in the Ghana Randomized Air Pollution and Health Study (GRAPHS): Ultrasound Capacity Building, Fetal Biometry Protocol Development, and Ongoing Quality Control
Source: JMIR Res Protoc. 2014 Dec 18;3(4):e77. doi: 10.2196/resprot.3797 (PMC4376157; doi:10.2196/resprot.3797)
Supplement: Supplementary file 1 [file resprot_v3i4e77_app1.pdf]

Ghana Randomized Air Pollution Health Study

ULTRASONOGRAPHER		Haruna
PARTICIPANT ID		BM00296S
DATE OF THE SCAN		30-Jul-13
EDD BY TRIAL OB		21-Nov-13

	Weeks	Days		Weeks	Days		AVERAGE
BPD	23	4		23	3		23 4/7
FL	23	3		23	6		23 5/7
TOTAL AVERAGE							23 5/7

Appendix 1: Representative Sample of Ultrasound Summary Report
